# Gastroschisis Simulation Model: Pre-surgical Management Technical Report

**DOI:** 10.7759/cureus.1109

**Published:** 2017-03-22

**Authors:** Orna Rosen, Robert M Angert

**Affiliations:** 1 Neonatology, Pediatrics, Children's Hospital at Montefiore, Montefiore Medical Center

**Keywords:** gastroschisis, delivery room, pre-surgical management, resuscitation, newborn, abdominal wall defect

## Abstract

This technical report describes the creation of a gastroschisis model for a newborn. This is a simple, low-cost task trainer that provides the opportunity for Neonatology providers, including fellows, residents, nurse practitioners, physician assistants, and nurses, to practice the management of a baby with gastroschisis after birth and prior to surgery. Included is a suggested checklist with which the model can be employed. The details can be modified to suit different learning objectives.

## Introduction

Gastroschisis is a congenital defect that occurs in 1:5000 live births [1]. The care of these newborns in the delivery room is of critical importance. Since the bowel is protruding outside of the abdominal cavity, there is a great risk of compromising the blood supply by twisting or by increasing mechanical pressure on it. There is a risk of infection by exposure of the gut to the outside air. Babies with gastroschisis are at a risk of dehydration and shock. The management of these patients is complex and requires technical skills, sound decision making, and anticipation of potential complications [2].

**Figure 1:**
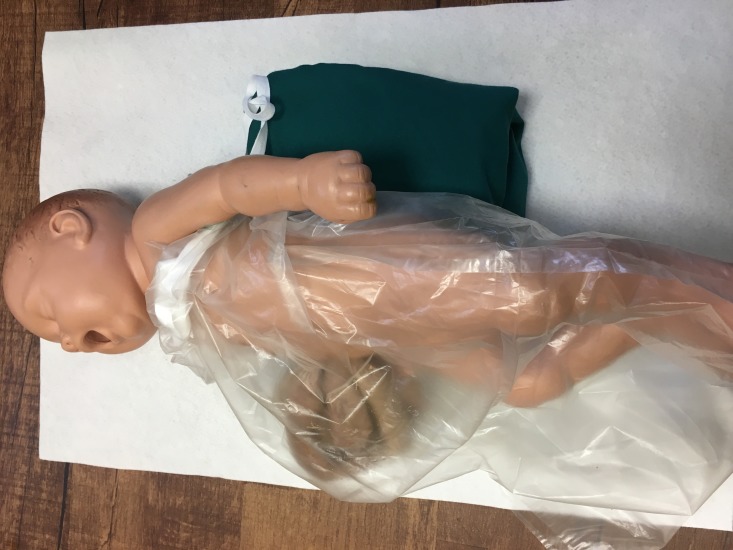
Simulation Model With Occlusive Bag in Place Babies with gastroschisis are placed in an occlusive bag up to their axilla and the cotton tape is cinched, protecting the baby from evaporative and thermal losses.

Neonates with gastroschisis are typically placed in a plastic bag or wrap. If a bag is used, the baby’s body is placed in the bag (legs first) up to the area just above the nipple line. The hands are left outside of the bag and then the string is pulled gently (Figure 1).

The baby is placed with the right side down to decrease the risk of compromise to the vascular supply of the gut.

In order to provide better care for a newborn with gastroschisis, we built an original task trainer. Our simulator is inexpensive to make, simple to assemble, and has a realistic appearance and feel. It is superior to other models due to its ease of assembly and readily available components [3].

We use this simulation-based model in our yearly boot camp which is a condensed course for critical neonatal intensive care unit (NICU) procedures, and during monthly simulation sessions that run throughout the year. Training can also be conducted in a just-in-time [4] method before a planned gastroschisis delivery.

## Technical report

**Figure 2:**
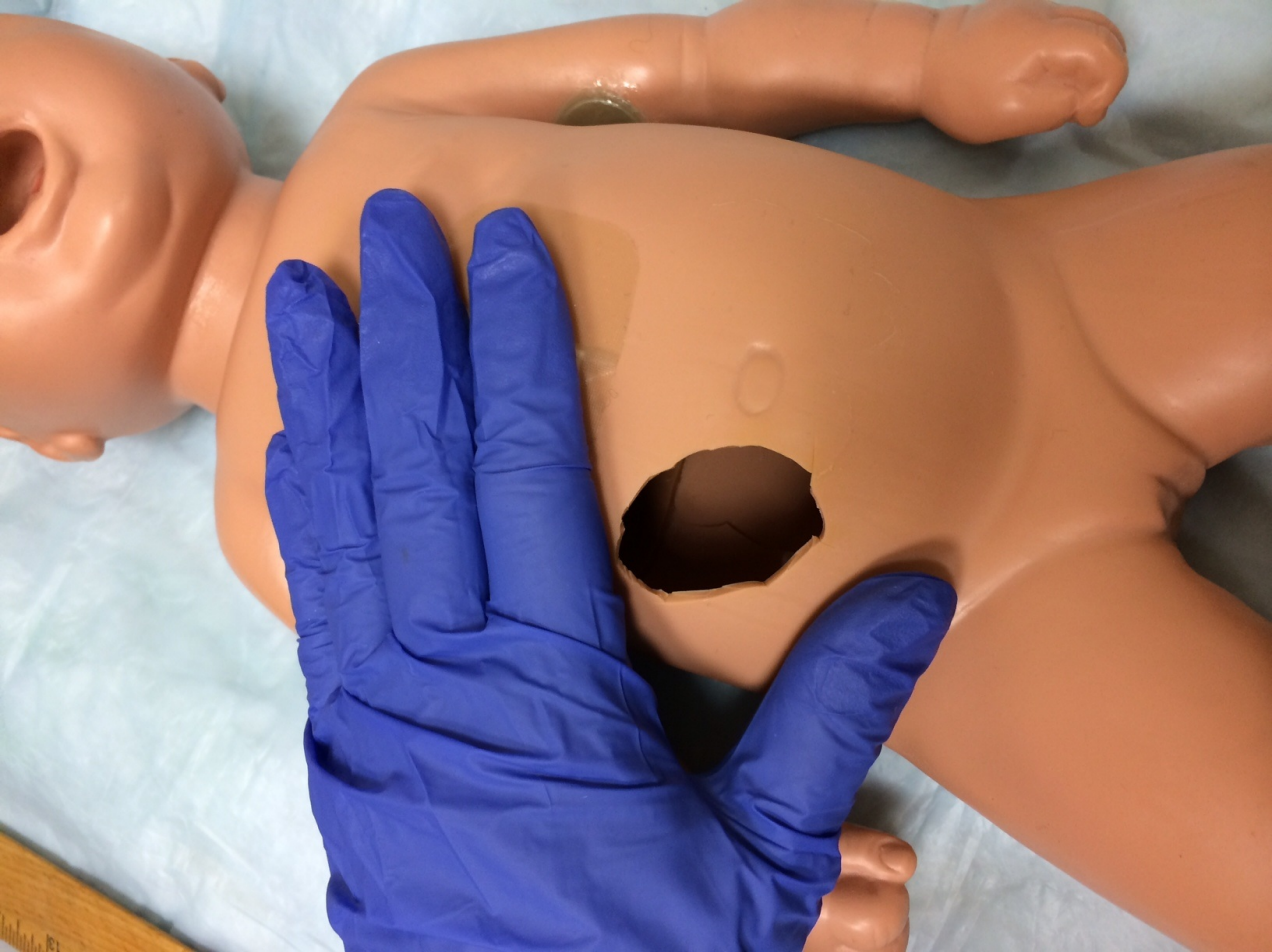
Manikin With Hole A 4-cm hole was made in the abdomen of the manikin.

We used a low-technology manikin (1978, Simulaids) but this model can be applied to most resuscitation trainers. To the right of the umbilicus, we carved a hole of 4 cm x 4 cm with a scalpel blade in order to insert the simulated intestines (Figure 2). This procedure damages the manikin and cannot be repaired. It is important to take this into account when choosing a suitable manikin.

**Figure 3:**
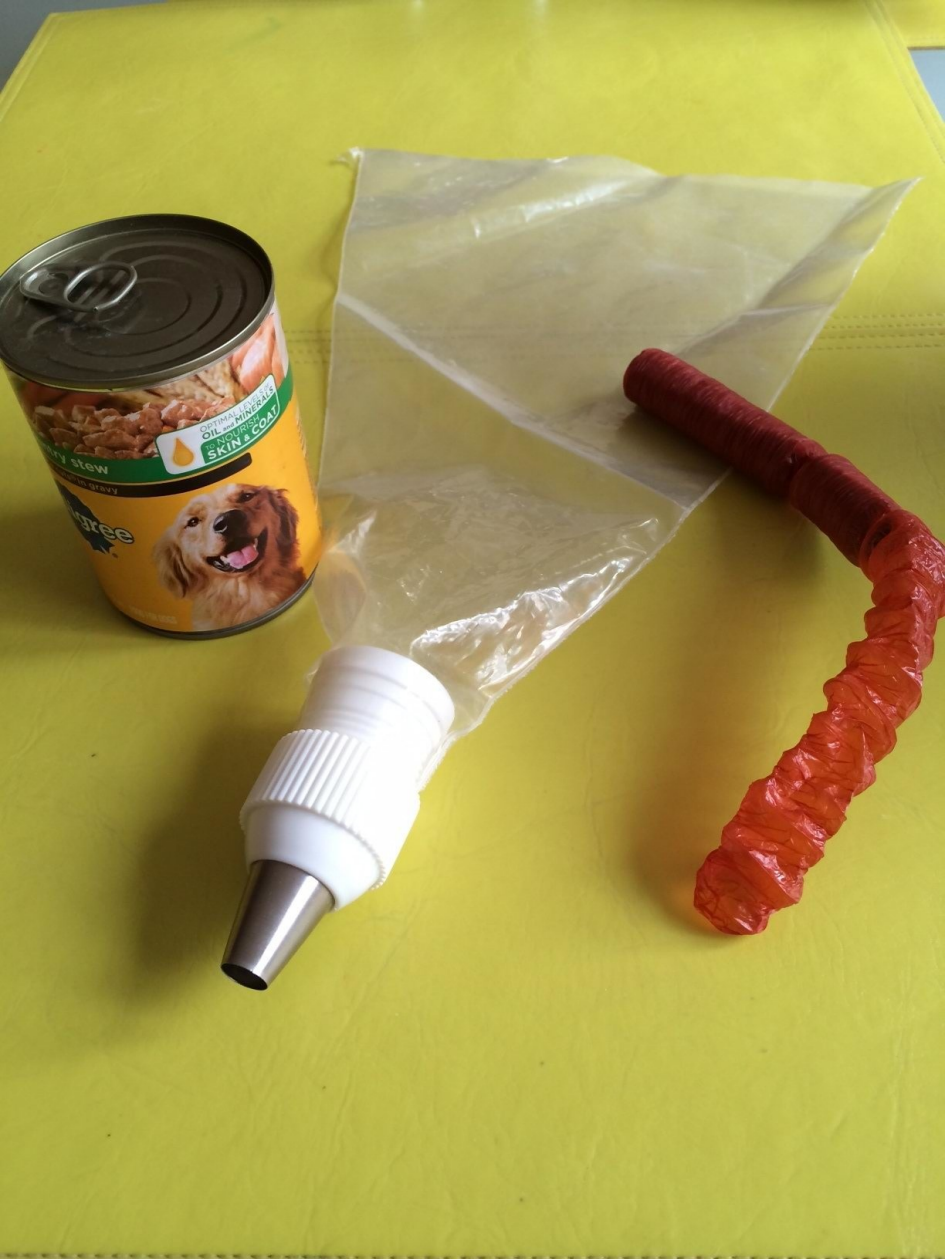
Materials for Making an Intestine We used a pastry piping device, sausage casing, and a can of dog food to create the simulated bowel.

The simulated intestines: We used edible snack stick collagen casing (21-mm diameter, cost $21.75 including shipping). For bowel contents, we used two cans of soft dog food. Dog food was chosen because the texture and color closely resembled the viable intestine. We tried gelatine but the color and texture were inadequate. We crushed the dog food in a food processor and used a 16-inch cake decorating piping bag with a tip diameter of 10 mm (Figure 3). The crushing can also be done with a fork or alternatively ground pet food could also be used.

**Figure 4:**
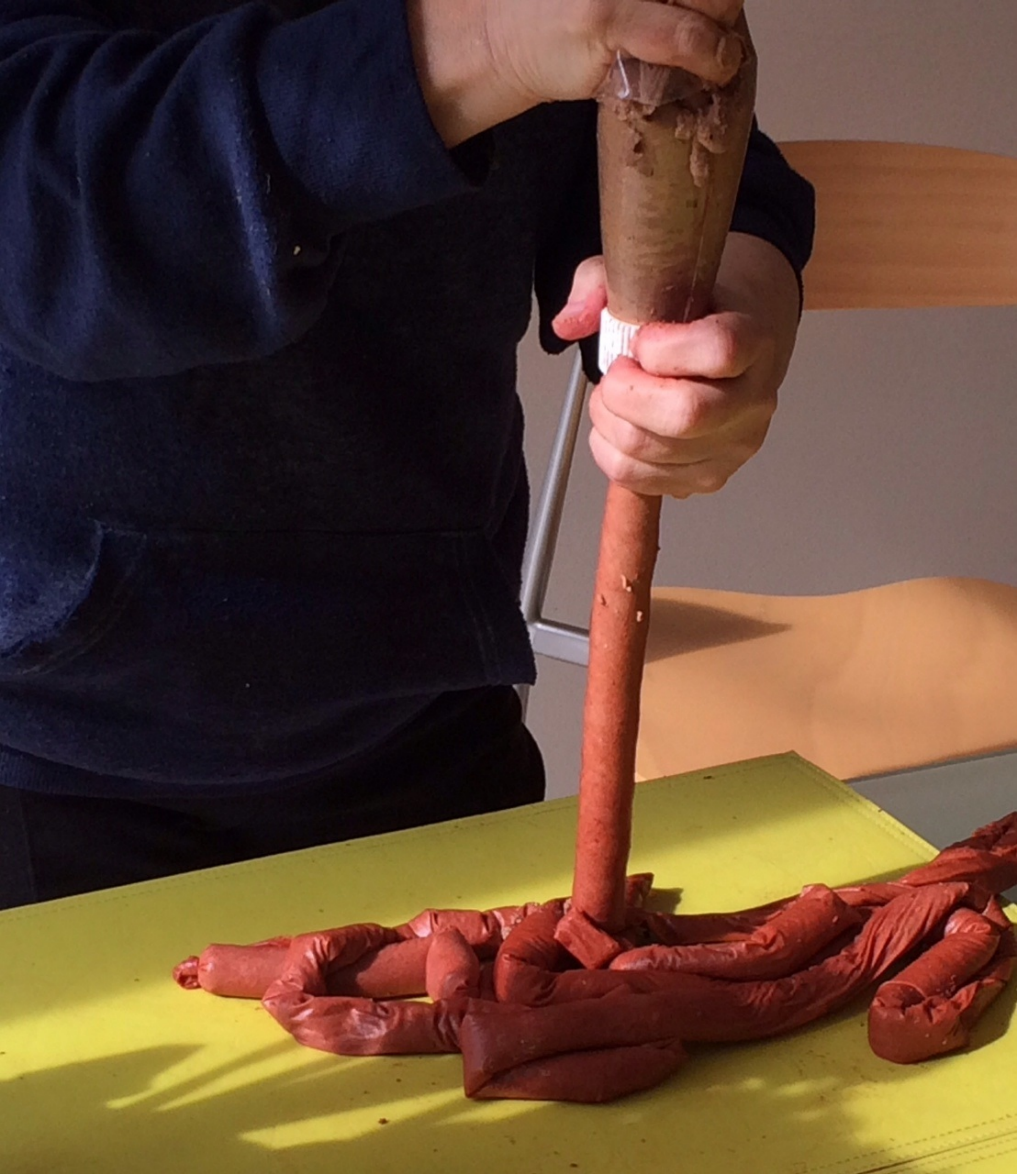
Making the Intestine The piping bag is held together with the sausage casing and the processed dog food is squeezed into the casing, working it manually to the distal knotted end.

The distal end of the 275-cm length segment of the sausage casing was knotted. We chose this length to simulate the length of the bowel of a full term baby, but less could be used to simulate a smaller gastroschisis. The dog food was transferred to the piping bag, the sausage casing was attached to the tip of the piping bag, and the contents were squeezed into the casing. From time to time, we stopped pipetting and pushed the contents further in by squeezing the casing with our hands. The casing was filled loosely so it could still be easily manipulated and the proximal end was tied in a knot (Figure 4).

**Figure 5:**
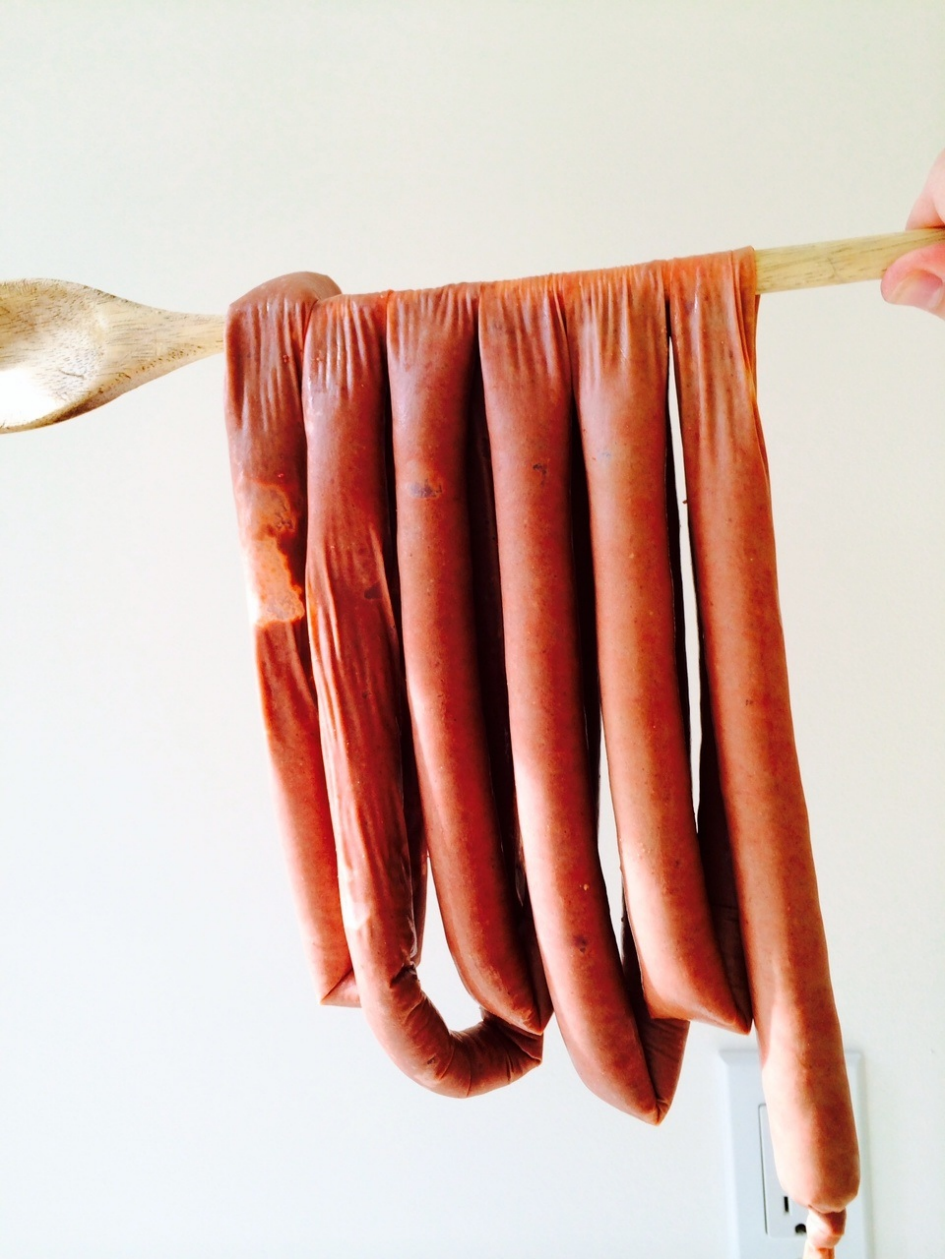
Loops of Simulated Intestine Loops of simulated intestine (25-cm long) were draped over a wooden spoon and then were tied together to make a shape similar to a "bouquet".

Assembly of the simulator: To create bowel loops, we took a 40-cm wooden spoon, coiled the filled casing around the spoon handle in loops of about 50 cm in length (25 cm on each side of the spoon), then threaded a 50-cm inch string instead of the spoon and tied it to create a "bouquet" of loops (Figure 5).

**Figure 6:**
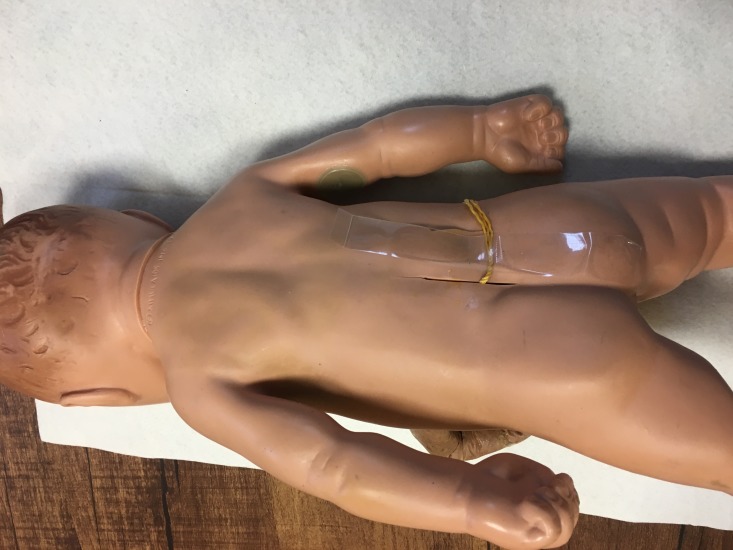
Securing Simulated Intestines Affixed to the Back The string holding together the loops of intestine are pulled through the back and held in place with a tape. The model is placed in prone position in this photograph to demonstrate the method of securement.

We inserted the “bouquet” into the hole in the abdominal wall of the doll, passing the string through a slit in the doll’s back and taping it to the back for a more secure attachment (Figure 6).

**Figure 7:**
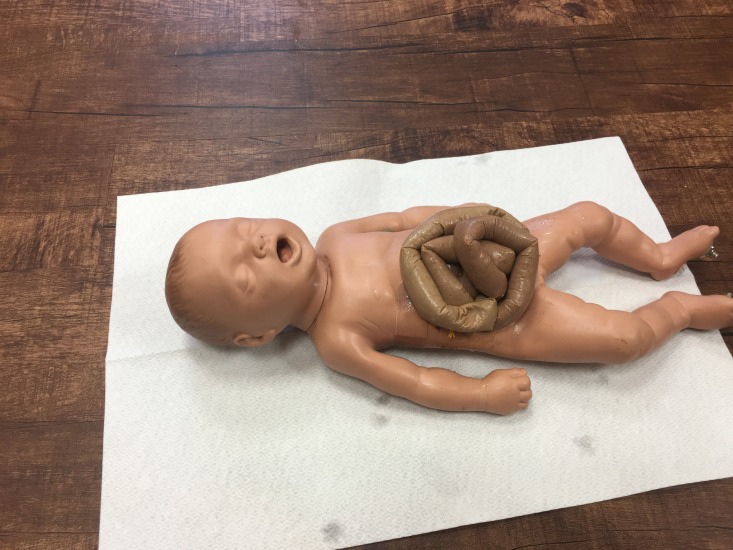
Manikin With Simulated Intestine in Place Simulated intestines in place and secured with the string protruding through the back and held in place with a tape.

The final product is shown below in the supine position, ready for use in simulation-based training (Figure 7).

After the use of this task trainer, the simulated intestine was stored in the freezer. By thawing it and refreezing it, we were able to reuse it 10 times, and we have plans to use it in the future as well. The doll was cleaned with chlorhexidine after each use.

### Learning Objectives

A. Trainees and providers will apply the knowledge of how to position and perform initial steps of resuscitation of newborns with gastroschisis.

B. Evaluate a baby with gastroschisis. Formulate and demonstrate a treatment plan with regard to respiratory management and gastric decompression.

C. Recognize and treat hypovolemia in an infant with gastroschisis (optional).

D. Recognize and treat bowel that has vascular compromise (optional).

E. Complete a scenario using the critical action checklist (below) that demonstrates the trainees' ability to evaluate a complex situation, design a treatment plan., and demonstrate it on a model.

Critical action checklist for Gastroschisis handling:

1. Ensure adequate supplies are gathered 2. Delegate tasks to team members 3. Place baby in the steri-drape 50 x 50 plastic bag (3M Health Care) 4. Attach pulse oximeter to the right hand 5. Suction and intubate the baby by avoiding positive pressure ventilation if respiratory failure is included in the scenario. 6. Secure endotracheal tube 7. Insert oropharyngeal replogle tube 8. Dry the upper part of the body 9. Attach a temperature probe if a prolonged stay in the delivery room is anticipated 10. Place baby with the right side down 11. Place an intravenous (IV) on baby’s upper arm 12. Transfer baby to the NICU 13. Upon admission to the NICU, check the baby’s vital signs and hydration status. Start the baby on IV fluids per unit protocol 14. Attach the replogle to low continuous suction 15. Check vital signs q15 minutes, replace any losses from the orogastric (OG) tube with electrolyte containing solutions as is customary to local practice 16. Consider administration of normal saline boluses in addition to maintenance fluids to keep normal vital signs and urine output

## Discussion

We have used this model more than 10 times for training scenarios and at national meetings. Our neonatal fellows, nurse practitioners, and physician assistants report better understanding and confidence in handling a baby with gastroschisis after practicing on the task trainer. Participants in the training scenarios commented: "The gastroschisis model was very good. It had a very life-like feel, just like a real bowel."  "It improved my confidence in handling this condition in the delivery room and the immediate pre-operative period." "Having been called to both real and simulation-based gastroschisis, it felt just like a real bowel."  "Using the model helped with positioning, and I knew just what to do and how to handle the bowel in a real situation." 

The scenarios can be varied depending on the experience level and learning objectives for the particular group. The complexity can be increased by adding complications that have occurred in clinical cases, such as a strangulation of the blood supply, hypotension from inadequate fluid administration, or hypothermia.

Positive pressure ventilation (PPV) is to be avoided when resuscitating a baby with an abdominal wall defect in order to prevent air from trapping in the intestines that could make the abdominal wall closure more difficult. This learning point can be added to the most routine scenario and can be brought up in the debrief. The scenario can be further modified to include silo placement which would include interprofessional training of surgical residents.

By employing this gastroschisis model, we are able to teach trainees to perform the actual procedures in a safe environment where team-based performance and communication skills can also be developed. This model for gastroschisis has the look and feel of a real intestine. This makes the experience more life-like or real. The simulation can be conducted in-situ or in a remote area from the delivery room and the NICU. This could allow assessment of the actual clinical environment for safety threats and increase the transfer of learning. The atmosphere is not meant to be threatening and care must be taken to establish ground rules for a positive learning environment. The debrief after the scenario is run by the trainees along with the trained faculty who act as facilitators. This guided group discussion helps diminish the tension and complete the learning objectives.

## Conclusions

This report contains detailed instructions and images that will help medical providers create their own models of a gastroschisis task trainer that is easy to assemble, inexpensive, reusable, and realistic. Using this model will improve the familiarity and performance of trainees who care for newborns with gastroschisis.
